# Genome-wide analysis of TPS family reveals kaurene synthase-like genes in *Isodon rubescens* f. lushanensis

**DOI:** 10.3389/fpls.2026.1937259

**Published:** 2026-09-11

**Authors:** Hao Yang, Jinlu Liu, Conglong Lian, Hongwei Yu, Le Zhao, Ni He, Yuanyuan Li, Ruohan Chen, Xiuyu Liu, Shujuan Xue, Xiaoya Sun, Lipeng Zhang, Lili Wang, Jingfan Yang, Yu Fu, Rui Ma, Bao Zhang, Lidan Ye, Suiqing Chen

**Affiliations:** 1 College of Pharmacy, Henan Key Laboratory of Chinese Medicine Resources and Chemistry, Henan University of Chinese Medicine, Henan, Zhengzhou, China; 2 Collaborative Innovation Center of Research and Development on the Whole Industry Chain of Yu-Yao, Henan, Hangzhou, China; 3 Co-Construction Collaborative Innovation Centre for Chinese Medicine and Respiratory Diseases by Henan & Education Ministry of China, Henan, Zhengzhou, China; 4 Institute of Bioengineering, College of Chemical and Biological Engineering, Zhejiang University, Zhejiang, Zhengzhou, China

**Keywords:** gene family analysis, *Isodon rubescens* (Hemsl.) Hara, *Isodon rubescens* f. lushanensis, terpene synthase, yeast heterologous expression

## Abstract

**Introduction:**

*Isodon rubescens* f. lushanensis, a typical form of *I. rubescens*, is characterized by its natural deficiency in oridonin and the presence of a unique ent-kaurane diterpenoid, lushanrubescensin.

**Methods:**

To elucidate the molecular basis underlying the distinct chemical profiles between *I. rubescens* f. lushanensis and *I. rubescens* (Hemsl.) Hara, we conducted a comprehensive analysis utilizing high-quality genomic and transcriptomic data of *I. rubescens* f. lushanensis.

**Results:**

Through a genome-wide survey, our study identified 83 terpene synthase (*TPS*) genes, including 13 *TPS*-C and 9 *TPS-e*/f subfamily genes, which are implicated in diterpenoid biosynthesis. By integrating conserved motif analysis, differential expression profiling (FPKM), and RT-qPCR validation, we successfully screened key candidate genes. Subsequent heterologous expression in *Saccharomyces cerevisiae* enabled the functional characterization of five diterpene synthase genes, specifically CPS and KSL enzymes, involved in the central modules of diterpenoid synthesis.

**Discussion:**

These findings not only expand the repertoire of known biosynthetic genes in *I. rubescens* but also offer valuable insights into the divergent biosynthetic pathways of oridonin and lushanrubescensin, paving the way for future metabolic engineering and synthetic biology studies.

## Introduction

*Isodon rubescens* (Hemsl.) Hara is a medicinal herb belonging to the family *Lamiaceae* and is primarily distributed in Henan and Shanxi provinces. In Henan Province, *I. rubescens* comprises three varieties and two forms. In botanical taxonomy, a “form” (forma, abbreviated as f.) is an infraspecific rank used to distinguish individuals with subtle but stable morphological or physiological characteristics The two forms examined in this study are *I. rubescens* (Hemsl.) Hara (hereafter designated *I. rubescens*-JY), originally collected from Jiyuan City in the Taihang Mountains, and *I. rubescens* f. lushanensis (hereafter designated *I. rubescens*-LS), originally collected from Lushan County in the Funiu Mountains (Gao and Li, 1986; Yang and Liu, 2020). These two forms exhibit distinct diterpenoid profiles: *I. rubescens*-JY is characterized by oridonin, whereas *I. rubescens*-LS produces lushanrubescensin (Chen et al., 2011). Both compounds belong to the *ent*-kaurane diterpenoid class and have been reported to possess anti-tumor activities (Chen et al., 2016; Lian et al., 2022). Their structural differences are primarily attributable to distinct downstream functional-group modifications. Lushanrubescensin contains four acetyl substituents, which may increase its lipophilicity relative to oridonin and highlight its potential pharmaceutical relevance (Wang, 1987; Zhao et al., 2007).

Bioactive diterpenoids, including oridonin and lushanrubescensin, are still predominantly obtained through plant extraction, which is constrained by limited natural resources and low extraction efficiency. The chemical synthesis of complex terpenoids is also challenging because of their structural complexity, stereochemical requirements, and environmental concerns. Synthetic biology provides a promising alternative for producing valuable terpenoids in microbial chassis, as demonstrated by the engineered production of paclitaxel, gastrodin and ferulic acid in *Saccharomyces cerevisiae* or *Escherichia coli* (Liu et al., 2026; Wang et al., 2026; Shang et al., 2025). However, the biosynthetic pathway of lushanrubescensin remains largely unsolved, limiting its sustainable production through metabolic engineering.

Previous studies have substantially advanced the understanding of diterpenoid biosynthesis in *I. rubescens*-JY. The diterpenoid pathway can be broadly divided into upstream precursor-supply reactions, central cyclization steps, and downstream tailoring reactions. Geranylgeranyl diphosphate (GGPP), generated through the cytosolic mevalonate (MVA) pathway or plastidial 2-*C*-methyl-D-erythritol 4-phosphate (MEP) pathway, is converted by class II copalyl diphosphate synthases (CPSs) into copalyl diphosphate (CPP) intermediates, which are subsequently cyclized by class I kaurene synthase-like (KSL) enzymes to generate diverse diterpene scaffolds. In *I. rubescens*-JY, several CPS and KSL enzymes have been functionally characterized, including *IrKSL5*, which catalyzes the formation of *ent*-kaurene, an early intermediate in the oridonin biosynthetic pathway (Jin et al., 2017). Recent chromosome-level genomic analysis further identified multiple CYP706 family proteins which catalyze early oxidative modifications of the *ent*-kaurene core during oridonin biosynthesis (Sun et al., 2023). Nevertheless, the TPS repertoire and the early cyclization steps leading to lushanrubescensin formation in *I. rubescens*-LS remain unexplored. To address this knowledge gap, we performed the first genome-wide analysis of the TPS family in *I. rubescens*-LS using genomic and transcriptomic resources. We further prioritized candidate diterpene synthase (diTPS) genes by integrating phylogenetic relationships, conserved-motif analysis, expression profiles, and co-expression associations, followed by functional validation in engineered yeast. By identifying and characterizing five CPS/KSL enzymes, this study establishes a molecular framework for the early cyclization steps of diterpenoid biosynthesis in *I. rubescens*-LS. These findings extend prior knowledge derived from *I. rubescens*-JY and provide a foundation for comparative analyses of diterpenoid-pathway diversification and for future identification of downstream CYP450s, acyltransferases, and other tailoring enzymes involved in lushanrubescensin biosynthesis.

## Materials and methods

### Plant materials

The seeds of two distinct chemotypes of *I. rubescens* were collected from their respective natural producing regions in Henan Province, China: *I. rubescens*-JY from Jiyuan City and *I. rubescens*-LS from Lushan City. These plant materials were formally identified by Professor Suiqing Chen from Henan University of Chinese Medicine.

For RT-qPCR analysis, 15 individual plants of each form were cultivated synchronously in an artificial climate chamber. The growth conditions were maintained at a constant temperature of 24–26 °C and a relative humidity of 60%, under a 16 h/8 h light/dark photoperiod with a light intensity of 120 µmol m^−2^s^−1^. Healthy roots and leaves from both forms were independently harvested, immediately flash-frozen in liquid nitrogen, and stored at -80 °C until RNA extraction. Three independent biological replicates were analyzed for each tissue type and form, with each biological replicate obtained from one individual plant. Three technical replicates were performed for each RT-qPCR reaction.

### Data sources

The T2T genome data of *I. rubescens*-LS were obtained from our previously published study (Yang et al., 2024) and have been deposited in the NCBI database under BioProject accession number PRJNA1089255. The full-length transcriptome data of *I. rubescens* used for gene annotation were also generated in our previous study and deposited in NCBI under BioProject accession number PRJNA1065080. The RNA-seq transcriptome data of *I. rubescens* were previously sequenced by our laboratory (Lian et al., 2025) and have been deposited in the Sequence Read Archive (SRA) of NCBI under BioProject accession number PRJNA910296. Protein sequences of *Arabidopsis thaliana* terpene synthase (TPS) genes were obtained from the *A. thaliana* Information Resource (TAIR) database (http://www.arabidopsis.org/).

### Identification and characteristics of *TPS*s in *I. rubescens*-LS

To identify TPS genes in *I. rubescens*-LS, 33 annotated TPS protein sequences from *A. thaliana* were retrieved from TAIR and used as queries for BLASTP searches against the predicted *I. rubescens*-LS proteome. BLASTP searches were conducted using BLAST version 2.5.0+ with an E-value cutoff of 1×10^-5^). Candidate proteins showing <30% amino acid identity or <50% query coverage relative to the *A. thaliana* TPS queries were excluded. The remaining candidates were examined for conserved TPS domains using Hidden Markov Model (HMM)-based searches. HMM profiles corresponding to the N-terminal TPS domain (PF01397) and C-terminal TPS domain (PF03936) were obtained from the Pfam database. Candidates were retained when they contained at least one TPS-associated domain; proteins containing both PF01397 and PF03936 were classified as full-length TPS candidates. TPS loci were extracted from the *I. rubescens*-LS genome using TBtools. Alternative isoforms and duplicated predictions originating from the same genomic locus were manually inspected, and one representative protein sequence was retained per locus. The final TPS set was obtained by intersecting BLASTP and HMM-search results.

The amino-acid length, molecular weight, theoretical isoelectric point, and total average hydropathicity of the predicted IrfTPS (*I. rubescens*-LS terpene synthases) proteins were calculated using ExPASy (https://web.expasy.org/protparam/). The subcellular localization was predicted using Cell-PLoc (http://www.csbio.sjtu.edu.cn/bioinf/Cell-PLoc/). Transmembrane helices and signal peptides were predicted using TMHMM-2.0 (https://services.healthtech.dtu.dk/service.php?TMHMM-2.0) and SignalP-3.0 (https://services.healthtech.dtu.dk/service.php?SignalP-3.0).

### Phylogenetic analysis of *TPS* genes

The protein sequences of *TPS* from *Salvia miltiorrhiza*, *S. hispanica*, *Arabidopsis thaliana*, and *Perilla frutescens* were obtained from NCBI (https://www.ncbi.nlm.nih.gov/). All protein accessions are listed in **Supplementary Table 5**. Multiple sequence alignments were performed using the MUSCLE method, and the phylogenetic tree was constructed using the neighbor-joining (NJ) method with 1000 bootstrap replications, employing the TBtools software. The resulting tree was visualized using the Chiplot online tool (https://www.chiplot.online/). The NJ tree was used primarily to classify TPS candidates and identify their relationships with previously characterized TPS proteins.

### Characteristics and gene expression levels of TPS-c and TPS-e/f family members

Members of the TPS-c and TPS-e/f gene families were subjected to multiple sequence alignment and conserved motif analysis using Jalview software (Jiang et al., 2019; Waterhouse et al., 2009). Proteins lacking the characteristic catalytic motifs required for class II or class I diterpene cyclase activity were excluded from subsequent functional-candidate screening. For phylogenetic placement, the *TPS* gene of *Physcomitrium patens* (XP_024380398.1) was used as an outgroup, and functionally characterized *TPS* genes from *I. rubescens* were used as reference proteins (**Supplementary Table 6**). The phylogenetic tree was constructed using TBtools and visualized using Chiplot (https://www.chiplot.online/).

Gene-expression levels were derived from the previously deposited RNA-seq dataset and expressed as fragments per kilobase of transcript per million mapped reads (FPKM) (Hayashi et al., 2006; Mortazavi et al., 2008). FPKM values were used for heatmap visualization and preliminary prioritization of candidate genes because the original RNA-seq dataset was processed using this normalization metric. FPKM values were not considered independent proof of differential expression or gene function. Candidate genes were further evaluated by RT-qPCR and heterologous functional assays.

### Validation of the differentially expressed genes by RT-qPCR

Five genes were selected for independent RT-qPCR validation of the RNA-seq expression patterns. In addition, 18 upstream diterpenoid-pathway genes and seven candidate diterpene synthase genes were analyzed to examine tissue-associated expression patterns. Total RNA was extracted from *I. rubescens* tissues using the FastPure Universal Plant Total RNA Isolation Kit (Vazyme, Nanjing, China), according to the manufacturer’s instructions. RNA quality and purity were verified using NanoDrop spectrophotometry (A_260_/A_280_ > 1.8; A_260_/A_230_ > 2.0) and 1.5% agarose gel electrophoresis. First-strand cDNA was synthesized from 1 μg of total RNA using HiScript III RT SuperMix for qPCR (+gDNA wiper) (Vazyme, Nanjing, China). Gene-specific primers were designed using the PrimerQuest Tool (**Supplementary Table 1**). RT-qPCR reactions were performed on a Light Cycler 96 instrument (Roche) with MonAmp SYBR Green qPCR Mix (Monad, Suzhou, China). The *actin* gene (GenBank: OP321409) was used as an internal reference because of its stable expression across all tested tissues in preliminary stability evaluation (coefficient of variation <5%). Relative expression levels were calculated using the 2^-ΔΔCt^ method based on three biological replicates, each with three technical replicates.

### Yeast strain construction

*The S. cerevisiae* strain YXWP-120, which features an enhanced supply of geranylgeranyl diphosphate (GGPP), served as the parental strain for constructing all engineered strains in this study (**Supplementary Table 2**). Key genes involved in the diterpenoid biosynthetic pathway (*CPS* and *KSL*) were codon-optimized for *S. cerevisiae* and synthesized by Qingke Biotechnology Co., Ltd. (**Supplementary Table 3**). Engineered strains were constructed using a decentralized metabolic pathway integration strategy facilitated by the pUMRI (marker recyclable integrative) plasmid system (Xie et al., 2015; Song et al., 2017). This system is based on the Cre/loxP recombination mechanism, allowing targeted genomic integration and subsequent recycling of the KanMX selection marker (Xie et al., 2014). Genes were assembled into pUMRI vectors and sequentially integrated into the yeast genome at predefined loci. Following each integration step, transformants were selected on YPD medium supplemented with 200 µg/mL geneticin (G418). All constructs were verified by colony PCR and Sanger sequencing before functional characterization. The expression of the integrated genes was controlled using a modified *GAL* regulatory system to achieve inducible and coordinated expression of the pathway enzymes.

### Cultivation in shaking flasks

The engineered yeast strains were pre-cultured in 5 mL YPD (1% yeast extract, 2% peptone, and 2% glucose) at 30 °C, 220 rpm for 24 h. The pre-cultures were then inoculated to an initial OD_600_ of 0.05 in 200 mL of fresh YPD medium. After 24 h of incubation, dodecane (1%, v/v) was added to the culture medium, and the culture was continued for 4 days.

### GC-MS identification of yeast metabolites

The assay was performed using a Trace 1310 series GC equipped with a TSQ8000 MS detector (Thermo Fisher Scientific Co. Ltd.) and a TR-5 MS capillary column (30 m×0.25 mm i.d.; DF = 0.25 μm; Thermo Fisher Scientific). Separation of the injected sample (1 μL) was achieved at a helium flow rate of 1 mL·min^-1^ with a temperature program of 1 min at 120 °C, followed by a gradient from 120 °C to 250 °C at 10 °C per minute. Commercial standards were not available for *ent*-atiserene, *ent*-kaurene, or miltiradiene. Therefore, reference yeast strains expressing previously characterized diterpene synthase combinations—CPS4FB + KSL4FB, CPS4FB + KSL5FB, and CPS1FB + KSL1FB—were constructed or used as reference strains for *ent*-atiserene, *ent*-kaurene, and miltiradiene, respectively, according to the established enzyme functions reported by (Jin et al., 2017). Metabolites were assigned by comparison of retention times and mass spectra with authentic standards, where available, or with reference strains producing established diterpene products.

### Molecular docking analysis of KSL enzymes with *ent*-CPP and normal-CPP

To explore the potential structural basis of substrate recognition by KSL homologs, molecular-docking simulations were performed using *ent*-CPP and normal-CPP as ligands. Before docking, protein structures were selected from the RCSB Protein Data Bank. Water molecules and non-essential ligands were removed, polar hydrogen atoms were added, and partial atomic charges were assigned using AutoDockTools. The ligand structures of *ent*-CPP and normal-CPP were retrieved from the PubChem database. Docking simulations were carried out using AutoDock Vina 1.2.5. The docking grid for each protein was centered on the predicted catalytic pocket, as defined by conserved active-site residues. The resulting binding poses were analyzed using Discovery Studio 2019 and PyMOL 2.6 to generate two-dimensional interaction diagrams and three-dimensional binding models. The AutoDock Vina docking score was used to evaluate the predicted ligand-receptor interactions. These criteria were applied consistently to all docking complexes.

## Results

### Identification and characteristics of IrfTPS family in *I. rubescens-LS*

Based on the results of the HMM search and BLASTP, a total of 83 Irf*TPS*s (*I. rubescens*-LS terpene synthases) were identified in the *I. rubescens*-LS genome (**Supplementary Table 4**). The physicochemical properties of the predicted IrfTPS proteins were then characterized. The protein lengths varied substantially, ranging from 135 (IrfTPS50) to 1578 (IrfTPS80) amino acids. The predicted molecular weights ranged from 15.49 to 181.08 kDa, while the theoretical isoelectric points (pI) ranged from 4.47 (IrfTPS77) to 7.93 (IrfTPS83), indicating considerable structural diversity within the IrfTPS family. Subcellular localization prediction suggested that most IrfTPS proteins are localized in the cytoplasm or chloroplast, consistent with the compartmentalized biosynthesis of terpenoids in plants. Notably, only IrfTPS61 was predicted to localize to the endoplasmic reticulum. No canonical transmembrane helices or N-terminal signal peptides were predicted for the identified IrfTPS proteins, suggesting that most members are likely soluble enzymes.

### Phylogenetic analyses and sequences analysis of IrfTPSs

**Figure 1:**
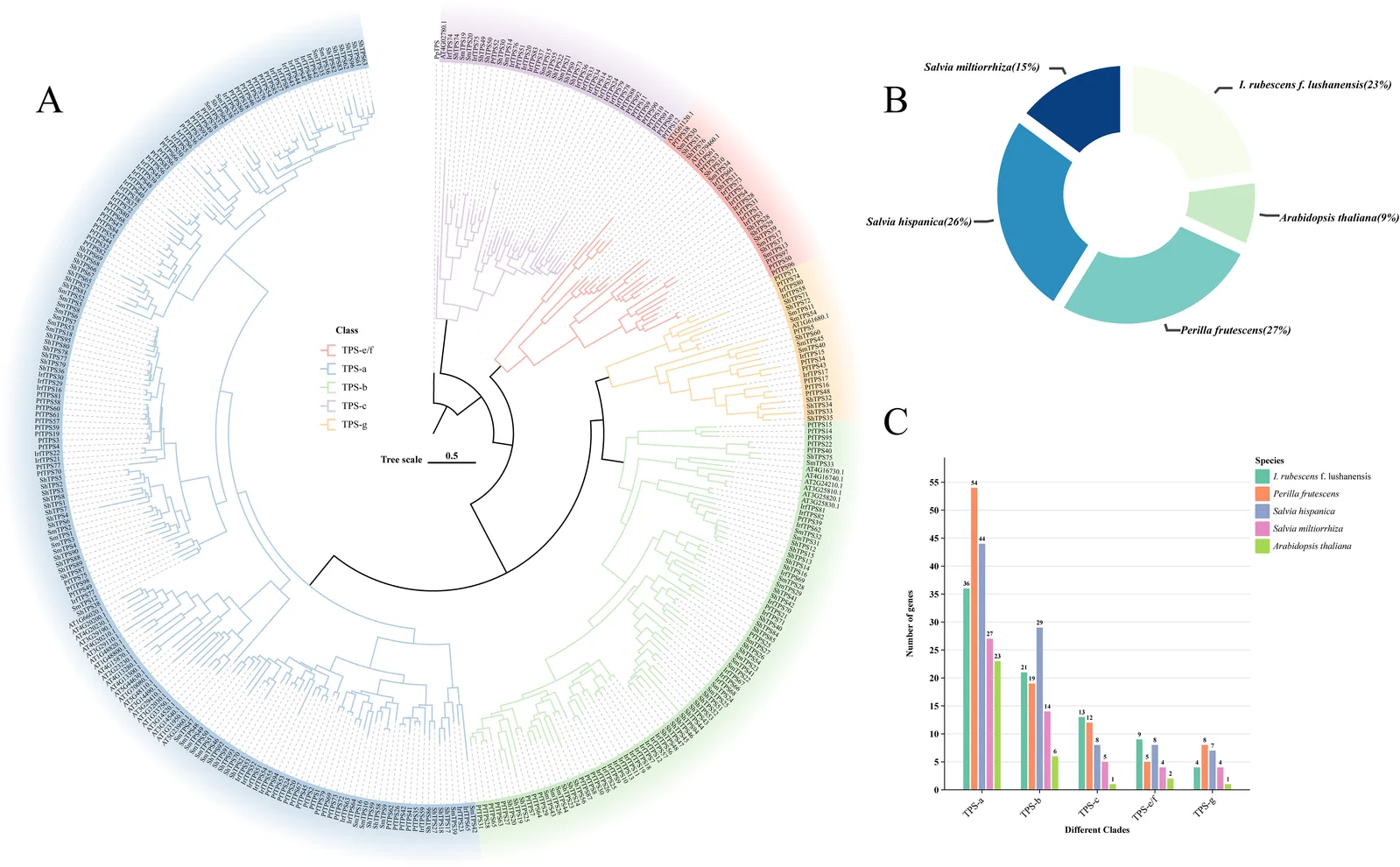
Phylogenetic classification and subfamily distribution of TPSs in *I. rubescens*-LS. **(A)** Neighbor-Joining phylogenetic tree of 364 TPSs from *I. rubescens*, *S. miltiorrhiza*, *S. hispanica*, *A. thaliana*, and *P. frutescens*. Branches are color-coded to distinguish five canonical TPS subfamilies (TPS-a, TPS-b, TPS-c, TPS-e/f, TPS-g). The scale bar represents amino acid sequence divergence. **(B)** Numbers and proportions of TPS genes from each species included in the phylogenetic analysis. **(C)** Distribution of TPS genes among the five TPS subfamilies in *I. rubescens*-LS.

To classify *TPS* genes in *I. rubescens*-LS, a phylogenetic analysis was performed using the TPS classification framework established in *A. thaliana* (Hu et al., 2019). A total of 281 TPS proteins from *S. miltiorrhiza* (54), *S. hispanica* (96), *A. thaliana* (33), and *P. frutescens* (98) were included (**Figures 1A, B**; **Supplementary Table 5**). Using bifunctional diterpene synthases from *Physcomitrium patens* as an outgroup (Pelot et al., 2017), a phylogenetic tree was constructed using the neighbor-joining (NJ) method. The IrfTPSs proteins were classified into five subfamilies: TPS-a, TPS-b, TPS-c, TPS-e/f, and TPS-g. Of the 83 genes, 36 belonged to TPS-a, 21 to TPS-b, 13 to TPS-c, 9 to TPS-e/f, and 4 to TPS-g (**Figure 1C**). The predominance of TPS-a subfamily genes is consistent with the extensive diversification of sesquiterpene synthases reported in other Lamiaceae species, suggesting an expansion of specialized terpenoid metabolism during the evolution of *I. rubescens*.

### Gene expression analysis of *TPS*-c and *TPS*-e/f in *I. rubescens*-LS

Diterpene synthase genes are mainly distributed in the TPS-c and TPS-e/f gene families. Class *II* diterpene cyclases, including CPSs, typically contain a conserved DXDD motif that initiates protonation-dependent cyclization, whereas class *I* diterpene synthases, including KSLs, possess a DDXXD motif that coordinates divalent metal ions and promotes substrate ionization. In the present analysis, the DDXXD motif was identified in the TPS-e/f candidates, whereas the DXDD motif was present in most TPS-c candidates; IrfTPS74, IrfTPS20, and IrfTPS35 lacked the expected complete class *II* catalytic motif and were excluded from the functional-candidate set (**Supplementary Figure 1**).

**Figure 2:**
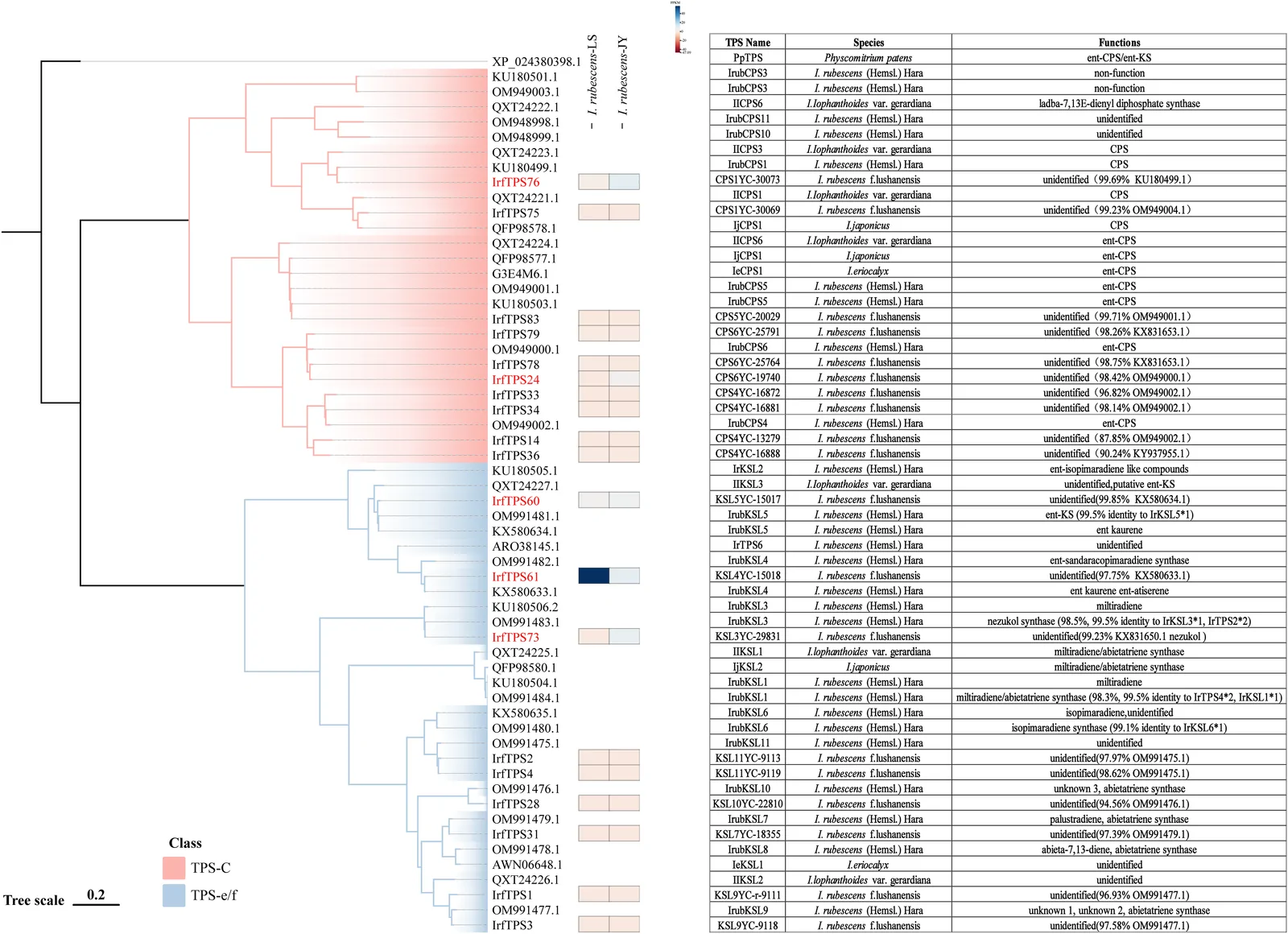
Gene expression levels of TPS-c and TPS-e/f candidates from *I. rubescens*-LS. The left panel shows a phylogenetic tree containing candidate TPS proteins from *I. rubescens*-LS and functionally characterized diterpene synthase references from *Isodon* species. Pink and blue branches indicate TPS-c and TPS-e/f clades, respectively. The accompanying table lists gene identifiers, source species, and reported catalytic products of the reference enzymes. Heatmap columns show FPKM-normalized transcript abundance in root and leaf RNA-seq samples from *I. rubescens*-LS and *I. rubescens*-JY; darker colors indicate higher transcript abundance. FPKM values were used for visualization and candidate prioritization. The five genes showing striking inter-chemotype expression divergence were selected for yeast-based functional assays, indicated by red fonts.

After excluding proteins lacking conserved catalytic motifs, 10 type *II CPS* genes and 9 type *I KSL* genes were retained for further analysis (**Figure 2**). Based on their phylogenetic relationship with functionally characterized *diTPS* genes from *Isodon* (**Supplementary Table 6**), the type *II* genes were designated *CPS1YC-30073*, *CPS1YC-30069*, *CPS5YC-20029*, *CPS6YC-25791*, *CPS6YC-25764*, *CPS6YC-19740*, *CPS4YC-16872*, *CPS4YC-16881*, *CPS4YC-13279*, and *CPS4YC-16888*. The type *I* genes were designated *KSL5YC-15017*, *KSL4YC-15018*, *KSL3YC-29831*, *KSL11YC-9113*, *KSL11YC-9119*, *KSL10YC-22810*, *KSL7YC-18355*, *KSL9YC-r-9111*, and *KSL9YC-9118*.

Candidate genes for functional assays were prioritized using multiple criteria: phylogenetic proximity to characterized *Isodon* diTPSs, retention of intact catalytic motifs, distinct transcript-abundance patterns between the two forms, tissue-preferential expression confirmed by RT-qPCR, and co-expression associations with other diterpenoid-pathway genes. RNA-seq expression profiles, presented as FPKM heatmaps, showed visually distinct transcript-abundance patterns for *CPS1YC-30073*, *CPS6YC-19740, KSL5YC-15017*, *KSL4YC-15018*, and *KSL3YC-29831* between *I. rubescens-*LS *and I. rubescens-*JY (**Figure 2**). These patterns were used for initial candidate prioritization and were subsequently evaluated by RT-qPCR and yeast-based functional assays.

### Co-expression analysis of diterpenoid synthesis pathway genes in *I. rubescens*

Previous studies have shown that diterpenoid biosynthetic genes in *I. rubescens* exhibit tissue-dependent expression patterns (Sun et al., 2023). Therefore, roots and leaves from *I. rubescens-*LS and *I. rubescens-*JY were analyzed by RT-qPCR. Expression profiles were assessed for 18 upstream genes in the diterpene synthesis pathway, along with two previously characterized *CPS* genes (*CPS4FB* and *CPS1FB*), two *KSL* genes (*KSL5FB* and *KSL1FB*) from *I. rubescens-*JY (Jin et al., 2017), and the five selected *I. rubescens-*LS diTPS candidate genes. Predicted structural comparisons indicated differences in domain architecture and overall predicted tertiary conformation between KSL9YC-9118/KSL9YC-r-9111 and the JY homolog KSL9FB (**Supplementary Figures 3B**, **S4B**). These observations motivated inclusion of the KSL9 homologs in the expression analysis but were not considered direct evidence of altered enzymatic function.

**Figure 3:**
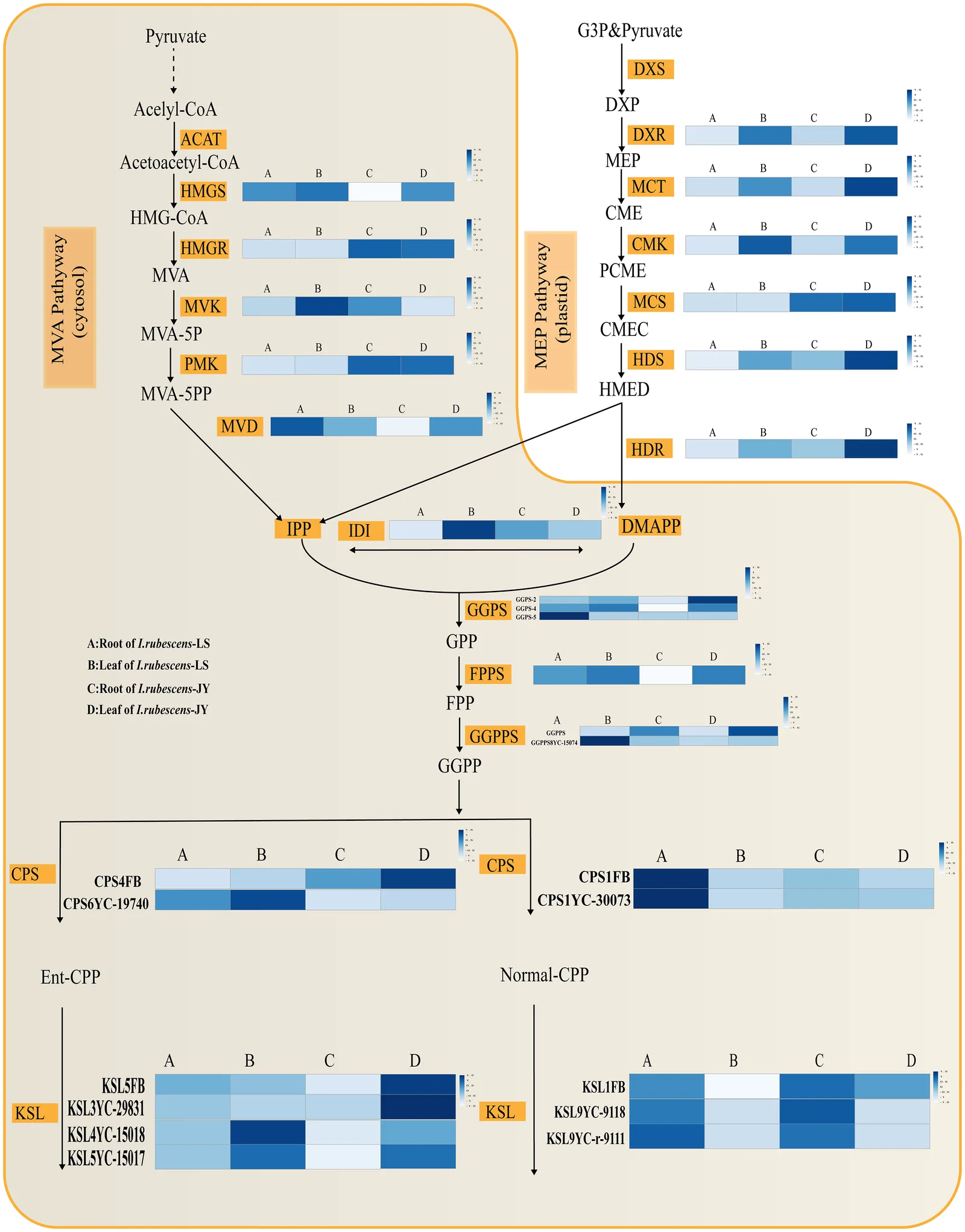
RT-qPCR expression profiles of genes associated with diterpenoid biosynthesis in roots and leaves of *I. rubescens*-LS and *I. rubescens*-JY. The heat map shows the relative expression levels of key diterpenoid synthesis pathway genes measured by RT-qPCR. Darker blue rindicates higher relative expression. A and B represent gene expression levels in the roots and leaves of *I. rubescens*-LS, and C and D represent gene expression levels in the roots and leaves of *I. rubescens*-JY. *CPS6YC-19740, KSL3YC-29831, KSL4YC-15018* and *KSL5YC-15017* showed leaf-preferential expression, whereas *CPS1YC-30073* showed root-preferential expression in *I. rubescens*-LS.

RT-qPCR analysis revealed tissue-associated expression patterns among the candidate genes (**Figure 3**). *CPS6YC-19740*, *KSL3YC-29831*, *KSL4YC-15018*, and *KSL5YC-15017* showed higher transcript abundance in leaves, whereas *CPS1YC-30073* showed higher transcript abundance in roots. These expression patterns suggest tissue-associated roles in diterpenoid metabolism but do not independently establish pathway function.

FPKM-based co-expression-network analysis showed that *CPS1YC-30073* and *KSL3YC-29831* displayed multiple co-expression associations with other diterpenoid-pathway-related genes in *I. rubescens*-LS (**Supplementary Figure 2**). This network information was used as supportive evidence for candidate prioritization. Co-expression relationships indicate correlated transcript patterns and do not, by themselves, demonstrate direct regulatory or biochemical interactions.

### Gene function verification by heterologous expression in yeast

**Figure 4:**
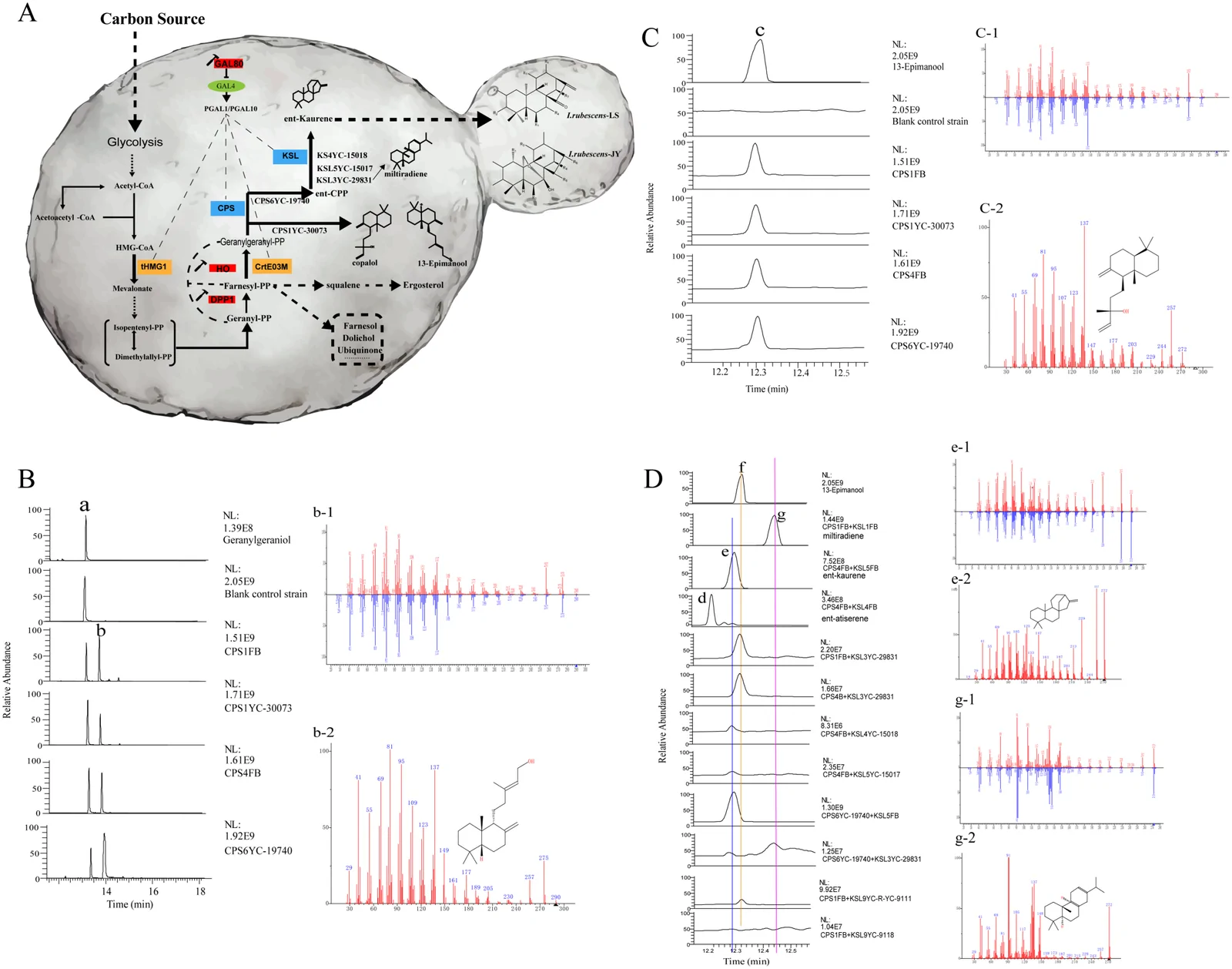
Functional characterization of candidate diterpene synthases from *I. rubescens*-LS in engineered *S. cerevisiae*. **(A)** Schematic overview of yeast strain construction for *CPS* and *KSL* gene function verification. Red labels indicate disrupted endogenous genes; yellow and blue labels indicate integrated heterologous genes. Dashed boxes identify target diterpenoid products analyzed by GC-MS. **(B)** GC-MS analysis of copalol-related products in yeast strains expressing *CPS* genes (*CPS1FB, CPS4FB, CPS6YC-19740*, and *CPS1YC-30073*) from *I. rubescens*-LS. Peak a, geranylgeraniol; peak b, copalol; b-1: Mirror-plot comparison of the detected compound spectrum (red, upward) and the corresponding NIST library reference spectrum (blue, downward). b-2: NIST library reference mass spectrum and chemical structure of the matched compound, with red peaks indicating relative ion abundance and blue labels denoting m/z values. **(C)** GC-MS analysis of 13-Epimanool-related products in yeast strains expressing *CPS* genes from *I. rubescens* -LS. Peak c, 13-epimanool; c-1: Mirror-plot comparison of the detected compound spectrum (red, upward) and the corresponding NIST library reference spectrum (blue, downward). c-2: NIST library reference mass spectrum and chemical structure of the matched compound, with red peaks indicating relative ion abundance and blue labels denoting m/z values. **(D)** GC-MS analysis of yeast strains co-expressing *CPS* and *KSL* genes from *I. rubescens*-LS. Peak d, *ent*-atiserene reference; peak e, *ent*-kaurene; peak f, 13-epimanool reference; peak g, miltiradiene. Panels e-1 and g-1: Mirror-plot comparison of the detected compound spectrum (red, upward) and the corresponding NIST library reference spectrum (blue, downward).; panels e-2 and g-2: NIST library reference mass spectrum and chemical structure of the matched compound, with red peaks indicating relative ion abundance and blue labels denoting m/z values. All heterologous genes were integrated at the *HO* locus of *S. cerevisiae* YXWP-120. The blank control denotes the parental strain carrying an empty vector integrated at the *HO* locus. Red and blue traces represent the mass spectra of the detected sample peak and the corresponding standard/reference peak, respectively.

To validate the functions of selected diTPS candidates, leaf-preferential genes (*CPS6YC-19740*, *KSL3YC-29831*, *KSL4YC-15018*, *and KSL5YC-15017)* and the root-preferential gene *CPS1YC-30073* were heterologously expressed in *S. cerevisiae.* Each gene was individually cloned into the integrative plasmid pUMRI-10 and introduced into the engineered yeast strain YXWP-120, which was previously optimized for enhanced GGPP production (Xie et al., 2014) (**Figure 4A**).

Expression of *CPS6YC-19740 and CPS1YC-30073* enabled the detection of copalol and 13-epimanool-related products in the engineered yeast strains (**Figures 4B, C**). Because endogenous yeast phosphatases can hydrolyze CPP intermediates to the corresponding diterpene alcohols (Cao et al., 2010), detection of copalol and 13-epimanool was used as indirect evidence for CPS-mediated formation of CPP intermediates. Geranylgeraniol (GGOH) was detected in all strains (**Figure 4B**), demonstrating an adequate intracellular supply of GGPP. Peak identities were assigned by comparison with authentic standards and/or reference strains, as described in the Methods. Peak-area profiles suggested different relative abundances of copalol and 13-epimanool among CPS-expressing strains. However, as no absolute calibration or enzyme-kinetic analysis was performed, these data are interpreted as semi-quantitative comparisons of relative product accumulation rather than direct measurements of catalytic efficiency. Copalol and 13-epimanool share the same molecular formula (C_20_H_34_O), but they differ in bond orientation and the hydroxyl-group and double-bond positions.

Previous studies have reported that CPS1FB and CPS4FB catalyze the formation of normal-CPP and *ent*-CPP, respectively (Jin et al., 2017). To characterize the function of *KSL* genes, leaf-enriched *KSL* genes were individually co-expressed with either *CPS4FB* or *CPS6YC-19740*, whereas root-enriched *KSL* genes were co-expressed with *CPS1FB*. Three KSL candidates (KSL5YC-15017, KSL4YC-15018, and KSL3YC-29831) produced diterpene products consistent with *ent*-kaurene formation (peak e) in the corresponding engineered strains. Notably, the strain expressing *KSL3YC-29831* additionally generated a product consistent with miltiradiene (peak g) (**Figure 4D**).

The identification of *ent*-kaurene-producing KSL activity in *I. rubescens*-LS supports the presence of early cyclization reactions shared with the oridonin-producing JY form. Previous work reported that the JY homolog KSL3 produces miltiradiene from normal-CPP (Jin et al., 2017), whereas KSL3YC-29831 in the present yeast assay produced products consistent with both *ent*-kaurene and miltiradiene. This difference suggests functional divergence between the homologs, although the observed product profile may also be influenced by the available CPP substrate, heterologous-host context, and relative enzyme expression.

Sequence and predicted-structure comparisons indicated that KSL3YC-29831 lacks the predicted *γ*-domain observed in its JY homolog KSL3FB (**Supplementary Figures 3A**, **S4A**). This structural difference may be associated with the distinct product profile, but direct causal evidence will require further structural and mutational analyses.Likewise, the *I. rubescens*-LS KSL9 homologs, *KSL9YC-r-9111* and *KSL9YC-9118*, did not generate products corresponding to the palustradiene-related activities reported for JY KSL9 (Sun et al., 2023). Predicted differences in domain architecture and tertiary conformation may contribute to these functional differences (**Supplementary Figures 3B**, **S4B**). Collectively, these results identify two CPS and three KSL enzymes with detectable activity in engineered yeast and establish a set of candidate diTPSs for the early cyclization steps of diterpenoid biosynthesis in *I. rubescens*-LS.

### Molecular docking to characterize the interaction of KSL enzymes with ent-CPP and normal-CPP

All five KSL3-type proteins showed strong binding affinity for ent-CPP: AFU61898.1-Salvia sclarea (−8.2 kcal/mol, hydrogen-bonded to ASP92), KSL3-FB (−8.8 kcal/mol, hydrogen-bonded to TYR-273, THR-318, HIS-356, GLN-355), KSL3a-FB (−9.4 kcal/mol, hydrogen-bonded to ASN-399, GLU-334), KSL3YC-29831 (−9.0 kcal/mol, hydrogen-bonded to ASN-358, THR-318), and TPS2-FB (−9.0 kcal/mol, hydrogen-bonded to THR-302, ASN-399, HIS-357). Extensive van-der-Waals and alkyl-type hydrophobic interactions further fix ent-CPP within the active-site cavity. These binding characteristics structurally support the ent-CPP recognition and ent-kaurene-forming activity of KSL3 homologs. KSL9 homologs exhibited divergent binding behaviors toward normal-CPP. UVC58051.1-KSL9 had weak binding (−6.2 kcal/mol, hydrogen-bonded to GLN331 、THR336 、LYS378), whereas KSL9YC-9118 (−8.0 kcal/mol, hydrogen-bonded to ASP-493, ARG-634, ASN-637) and KSL9YC-r-9111 (−9.7 kcal/mol, hydrogen-bonded to ARG-566, ASP-525) displayed strong binding. Compared with UVC58051.1-KSL9, KSL9YC-9118 and KSL9YC-r-9118 possessed distinct hydrogen-bond- and hydrophobic-residue profiles, which may alter substrate orientation in the catalytic pocket (**Supplementary Figure 5**).

## Discussion

The biosynthesis of bioactive ingredients in traditional Chinese medicine represents a critical frontier in pharmacological research, yet progress is frequently constrained by incomplete knowledge of the enzymes and pathways responsible for the formation of structurally complex natural products (Hu et al., 2019). Lushanrubescensin is a distinctive ent-kaurane diterpenoid that contains four acetyl substituents and displays greater lipophilicity than oridonin—the characteristic diterpenoid of *I. rubescens-*JY (Wang, 1987). These structural characteristics highlight its potential pharmaceutical relevance and motivate elucidation of its biosynthetic pathway. In the present study, we identified 22 diterpene synthase (diTPS) genes in *I. rubescens*-LS, comprising 13 type II copalyl diphosphate synthase (CPS) genes and 9 type I kaurene synthase-like (KSL) genes. By comparison, 26 diTPS genes, including 13 CPS and 13 KSL genes, have been reported in *I. rubescens*-JY. Thus, the two forms exhibit broadly comparable diTPS repertoires but differ in the composition of their KSL subfamilies. CPS enzymes from both forms generated normal-CPP and *ent*-CPP, whereas the characterized KSL enzymes from *I. rubescens*-LS produced two diterpene products, miltiradiene and *ent*-kaurene (**Supplementary Figure 6**). In contrast, KSL enzymes from *I. rubescens*-JY reportedly generated a broader range of products, including nezukol, palustradiene, abieta-7,13-diene, miltiradiene, isopimaradiene, *ent*-sandaracopimaradiene, *ent*-kaurene, and *ent*-atisirene (Jin et al., 2017; Sun et al., 2023). These observations indicate lower characterized KSL product diversity in *I. rubescens*-LS than in *I. rubescens*-JY. Furthermore, heterologous expression in *S. cerevisiae* functionally validated five diTPSs (CPS6YC-19740, CPS1YC-30073, KSL5YC-15017, KSL4YC-15018, KSL3YC-29831). This research Together, these results expand the currently available enzymatic toolkit for *Isodon* diterpenoid biosynthesis and provide a foundation for reconstructing the early cyclization steps of lushanrubescensin biosynthesis.

Comparative functional analysis indicated that the CPS complement and core cyclization activities are relatively conserved between *I. rubescens*-LS and *I. rubescens*-JY. Both forms possess CPS enzymes capable of producing stereoisomeric precursors (*ent*-CPP and normal-CPP), as supported by the formation of copalol and 13-epimanool in yeast strains expressing the newly identified *I. rubescens*-LS CPS genes. In contrast, the KSL family exhibited a reduced gene complement and a narrower experimentally characterized product spectrum in *I. rubescens*-LS. *I. rubescens*-LS contains 9 functional KSL genes, compared to the 13 KSL genes documented in *I. rubescens*-JY (Han et al., 2003) (**Supplementary Figure 7**). This difference may contribute to the distinct diterpenoid profiles of the two forms, although causal links between KSL gene-copy variation and chemotypic divergence require further investigation.

Sequence and domain-architecture comparisons further suggest possible functional diversification among KSL homologs. For instance, KSL3YC-29831 in *I. rubescens*-LS lacks the predicted γ-domain present in its JY homolog, KSL3FB (Sun et al., 2023). Whereas KSL3FB was reported to produce miltiradiene from normal-CPP, KSL3YC-29831 produced products consistent with both miltiradiene and *ent*-kaurene in the yeast assay. This association between domain architecture and product profile raises the possibility that structural variation contributes to functional diversification among KSL homologs. Independent loss of the γ-domain has been reported during plant diterpene synthase evolution, supporting the evolutionary plausibility of domain-architecture variation among TPS enzymes (Hillwig et al., 2011; Wang and Peters, 2023). Similarly, the *I. rubescens*-LS homologs of *I. rubescens*-JY KSL9 (KSL9YC-r-9111 and KSL9YC-9118) displayed predicted differences in domain architecture and tertiary conformation, particularly within the β-domain, and did not produce the palustradiene-related products reported for *I. rubescens*-JY KSL9 (Sun et al., 2023). Collectively, these observations suggest that variation in KSL gene complement, domain architecture, and protein sequence may contribute to diterpene scaffold diversity between the two forms.

To further investigate the potential structural basis of functional divergence, molecular-docking simulations were performed to compare the predicted binding modes of KSL homologs with *ent*-CPP and normal-CPP. Within the same docking workflow, the calculated docking scores were used as relative indicators of the favorability of the predicted enzyme-substrate binding poses (Madeddu et al., 2022). The simulations suggested that KSL3 homologs could accommodate *ent*-CPP and that KSL9 homologs could accommodate normal-CPP within their respective predicted active-site pockets. The top-ranked poses were stabilized by predicted hydrogen bonds, van der Waals contacts, and hydrophobic interactions involving alkyl-containing residues. Comparative docking analysis further indicated that amino-acid substitutions surrounding the predicted substrate-binding pocket may alter CPP orientation and local noncovalent interactions, thereby potentially contributing to the divergent product profiles observed among KSL paralogs. However, the docking results should be interpreted as model-dependent, hypothesis-generating evidence rather than direct measurements of substrate affinity, complex stability, or catalytic efficiency. Future structural studies, site-directed mutagenesis, and biochemical kinetic analyses will be necessary to verify the functional roles of the identified candidate residues. Nevertheless, these docking results provide a rational basis for prioritizing active-site residues for subsequent mutational analysis of CPP recognition and product specificity in KSL diterpene synthases.

The identification of functional *ent*-kaurene synthase activity (KSL5YC-15017, KSL4YC-15018, KSL3YC-29831) in *I. rubescens*-LS establishes *ent*-kaurene as a likely central intermediate in the biosynthetic routes to oridonin in *I. rubescens*-JY and lushanrubescensin in *I. rubescens*-LS. Because the two forms produce distinct oxidized and acetylated *ent*-kaurane diterpenoids, their metabolic divergence is likely to arise mainly from downstream tailoring reactions after *ent*-kaurene formation. Cytochrome P450 (CYP450) enzymes are expected to mediate sequential hydroxylation and oxidation reactions, while acyltransferases are likely to participate in the acetylation steps required for lushanrubescensin formation. In *I. rubescens*-JY, the tandemly duplicated enzymes *IrCYP706V2* and *IrCYP706V7* have been shown to catalyze early oxidative modifications of the *ent*-kaurene core during oridonin biosynthesis (Sun et al., 2023). Accordingly, comparative genomic and transcriptomic analyses of CYP450s—including CYP706V candidates—as well as candidate acyltransferases between *I. rubescens*-LS and *I. rubescens*-JY will be essential for identifying the downstream enzymes responsible for lushanrubescensin formation (Chen et al., 2016). The co-expression network generated in this study (**Supplementary Figure 2**) offers a preliminary basis for prioritizing such candidates, particularly genes co-expressed with the functionally validated CPS and KSL genes.

**Figure 5:**
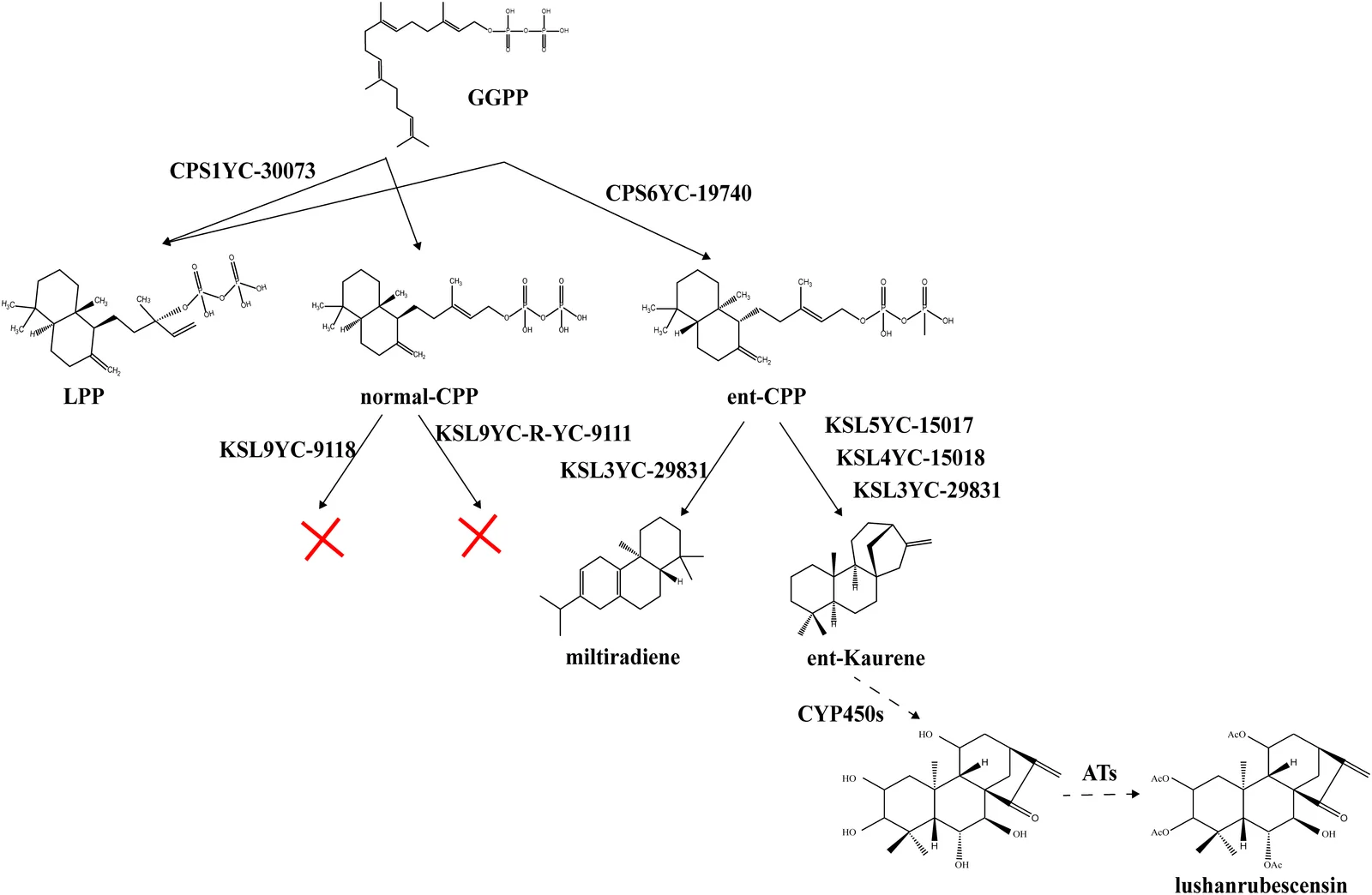
Proposed biosynthetic pathway of lushanrubescensin in *I. rubescens*-LS.Red crosses indicate that no detectable diterpene products were formed from normal-CPP by the two KSL9 enzymes. The experimentally validated CPS and KSL reactions are indicated by solid arrows. Putative downstream oxidation and acetylation steps catalyzed by CYP450s and acyltransferases are indicated by dashed arrows. CPS, copalyl diphosphate synthase; KSL, kaurene synthase-like enzyme; CYP450, cytochrome P450 monooxygenase; AT, acyltransferase.

Although this study establishes the TPS repertoire of *I. rubescens*-LS and functionally validates five CPS/KSL enzymes in yeast, the downstream enzymatic steps responsible for conversion of *ent*-kaurene to lushanrubescensin remain to be elucidated. In particular, the CYP450s, acyltransferases, and other potential tailoring enzymes involved in the oxidation and acetylation of the *ent*-kaurane scaffold have not yet been functionally characterized. Moreover, the association between KSL structural variation and catalytic divergence is inferred from sequence/domain comparisons and product profiles and will require further validation through structural and mutational analyses. Nonetheless, the engineered yeast strains capable of producing *ent*-kaurene provide a useful chassis for the functional screening of downstream CYP450s and acyltransferases. Future work integrating comparative multi-omics, co-expression-guided candidate selection, enzyme assays, and pathway reconstruction will be required to establish the complete lushanrubescensin biosynthetic pathway. An integrated pathway diagram summarizing the validated CPS/KSL enzymes, their products, and the proposed downstream CYP450- and acyltransferase-mediated reactions has been added as **Figure 5**. Collectively, this work establishes a genetic and enzymatic foundation for dissecting diterpenoid diversification in *I. rubescens* and for future microbial production of lushanrubescensin.

## Data Availability

The datasets presented in this study can be found in online repositories. The names of the repository/repositories and accession number(s) can be found in the article/**Supplementary Material**.
