# Supplementary material for: Genome-wide analysis of TPS family reveals kaurene synthase-like genes in Isodon rubescens f. lushanensis
Source: Front Plant Sci. 2026 Sep 11;17:1937259. doi: 10.3389/fpls.2026.1937259 (PMC13612420; doi:10.3389/fpls.2026.1937259)
Supplement: Supplementary file 1 [file DataSheet1.docx]

**Supporting Information**

**Genome-Wide Analysis of TPS Family Reveals Kaurene Synthase-Like Genes in *Isodon Rubescens* f. lushanensis**

Hao Yang^1,3,4^, Conglong Lian^1,3,4^, Hongwei Yu^2^, Le Zhao^1^, Jinlu Liu^1^, Ni He^2^, Yuanyuan Li^1^, Ruohan Chen^1^, Xiuyu Liu^1^, Shujuan Xue^1,3,4^, Xiaoya Sun^1,3,4^, Lipeng Zhang^1^, Lili Wang^1^, Jingfan Yang^1^, Yu Fu^1^, Rui Ma^1^, Bao Zhang^1^, Lidan Ye^2,*^ and Suiqing Chen^1,3,4*^

1 School of Pharmacy, Henan Key Laboratory of Chinese Medicine Resources and Chemistry, Henan University of Traditional Chinese Medicine, Henan, PR China.

2 Institute of Bioengineering, College of Chemical and Biological Engineering, Zhejiang University, Zhejiang, PR China.

3 Collaborative Innovation Center of Research and Development on the Whole Industry Chain of Yu-Yao，Zhengzhou 450046, Henan, PR China.

4 Co-Construction Collaborative Innovation Centre for Chinese Medicine and Respiratory Diseases by Henan & Education Ministry of China, Henan, PR China.

*Correspondence: Lidan Ye [(yelidan@zju.edu.cn),](mailto:(yelidan@zju.edu.cn),) Suiqing Chen [(chsq@hactcm.edu.cn)](mailto:(suiqingchen0371@163.com),)

†These authors contributed equally to this work.

**Supplementary Figures**

**Supplementary Figure S1. Conservative motif analysis of *TPS-c* and *TPS-e/f* in *I. rubescens*-LS**

**Supplementary Figure S2. Gene co-expression network of *I. rubescens*-LS.**

Grey lines (edges) represent co-expression correlations between genes (nodes). The degree of connectivity (number of connecting edges) reflects the centrality of a gene within the network, with highly connected nodes representing potential hub genes. The color intensity corresponds to the strength of the expression correlation.

**Supplementary Figure S3. Sequence comparison of candidate proteins**

(A) Comparison of KSL3 protein sequences

(B) Comparison of KSL9 protein sequences

**Supplementary Figure S4. Tertiary structure of KSL3 (A) and KSL9 (B) from *I. rubescens*-LS and *I. rubescens*-JY**

The pink region represents the DDXXD conserved motif, while the red region represents the DXDD(E) conserved motif.

**Supplementary Figure S5. Molecular docking analysis of KSL3s binding to ent‑CPP (A) and KSL9s binding to normal‑CPP (B)**

a-1,molecular docking of AFU61898.1‑*Salvia sclarea* binding to ent‑CPP; a-2,molecular docking of KSL3YC‑29831 binding to ent‑CPP; a-3,molecular docking of KSL3‑FB binding to ent‑CPP; a-4,molecular docking of KSL3a‑FB binding to ent‑CPP; a-5,molecular docking of TPS2‑FB binding to ent‑CPP.

b-1,molecular docking of UVC58051.1_KSL9 binding to normal‑CPP; b-2,molecular docking of KSL9YC‑9118 binding to normal‑CPP; b-3,molecular docking of KSL9YC‑r‑9111 binding to normal‑CPP.

**Supplementary Figure S6. Summary of key diterpene synthase enzymes in the biosynthesis of diterpenoid compounds in *I. rubescens*-JY**

**Supplementary Figure S7. Diterpenoid compounds synthesized in *I. rubescens*-JY**

**References**

Jin B. L., Cui G., Guo J., Tang J., Duan L., Lin H., et al. (2017) Functional diversification of kaurene synthase-like genes in *Isodon rubescens*. Plant physiology.174, 943-955. doi:10.1104/pp.17.00202

Xie W., L.Ye, Lv X., Xu H.,Yu H.(2015) Sequential control of biosynthetic pathways for balanced utilization of metabolic intermediates in *Saccharomyces cerevisiae*. Metabolic engineering.28, 8-18. doi:10.1016/j.ymben.2014.11.007
